# Triamcinolone acetonide acetate

**DOI:** 10.1107/S1600536811000316

**Published:** 2011-01-12

**Authors:** Xiao Lu, Gu-Ping Tang, Jian-Ming Gu, Xiu-Rong Hu

**Affiliations:** a Institute of Chemical Biology and Pharmaceutical Chemistry, Zhejiang University, Hangzhou, Zhejiang 310028, People’s Republic of China; b Chemistry Department, Zhejiang University, Hangzhou, Zhejiang 310028, People’s Republic of China

## Abstract

In the crystal structure of the title compound [systematic name: 2-(4b-fluoro-5-hy­droxy-4a,6a,8,8-tetra­methyl-2-oxo-2,4a,4b,5,6,6a,9a,10,10a,10b,11,12-dodeca­hydro-7,9-dioxa­penta­leno[2,1-*a*]phenanthren-6b-yl)-2-oxoethyl acetate], C_26_H_33_FO_7_, the mol­ecules are connected by inter­molecular O—H⋯O hydrogen bonds into an infinite supra­molecular chain along the *b* axis. The mol­ecular framework consists of five condensed rings, including three six-membered rings and two five-membered rings. The cyclo­hexa-2,5-dienone ring is nearly planar [maximum deviation = 0.013 (3) Å], while the cyclo­hexane rings adopt chair conformations. The two five-membered rings, *viz.* cyclo­pentane and 1,3-dioxolane, display envelope conformations.

## Related literature

For applications of triamcinolone acetonide in medicine, see: Barnes (1998 ▶); Buttgereit (2000 ▶); Uckermann *et al.* (2005 ▶). For the crystal structures of related triamcinolone acetonide acetates, see: Suitchlmezian *et al.* (2006 ▶); Jess & Näther (2006 ▶).
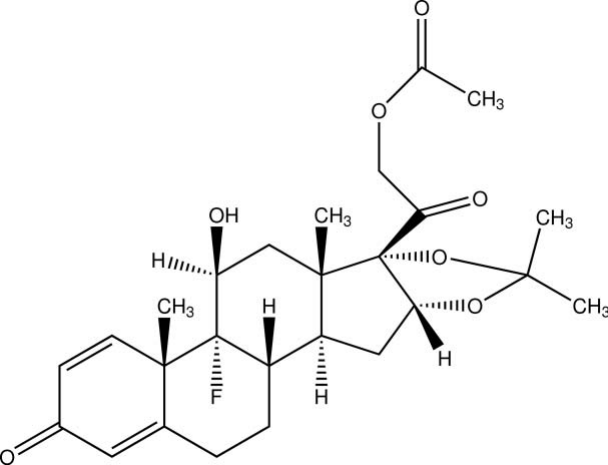


## Experimental

### 

#### Crystal data

C_26_H_33_FO_7_
*M* *_r_* = 476.52
Monoclinic, 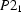

*a* = 7.5460 (7) Å
*b* = 14.8102 (4) Å
*c* = 11.5773 (3) Å
β = 109.905 (1)°
*V* = 1216.56 (12) Å^3^
*Z* = 2
Mo *K*α radiation
μ = 0.10 mm^−1^
*T* = 296 K
0.38 × 0.33 × 0.26 mm

#### Data collection

Rigaku R-AXIS RAPID/ZJUG diffractometer
9347 measured reflections
2216 independent reflections
1866 reflections with *I* > 2σ(*I*)
*R*_int_ = 0.027

#### Refinement

*R*[*F* ^2^ > 2σ(*F* ^2^)] = 0.035
*wR*(*F* ^2^) = 0.100
*S* = 1.00
2216 reflections
308 parameters
1 restraint
H-atom parameters constrained
Δρ_max_ = 0.18 e Å^−3^
Δρ_min_ = −0.21 e Å^−3^

### 

Data collection: *PROCESS-AUTO* (Rigaku, 2006 ▶); cell refinement: *PROCESS-AUTO*; data reduction: *CrystalStructure* (Rigaku, 2007 ▶); program(s) used to solve structure: *SHELXS97* (Sheldrick, 2008 ▶); program(s) used to refine structure: *SHELXL97* (Sheldrick, 2008 ▶); molecular graphics: *ORTEP-3 for Windows* (Farrugia, 1997 ▶); software used to prepare material for publication: *WinGX* (Farrugia, 1999 ▶).

## Supplementary Material

Crystal structure: contains datablocks global, I. DOI: 10.1107/S1600536811000316/xu5133sup1.cif

Structure factors: contains datablocks I. DOI: 10.1107/S1600536811000316/xu5133Isup2.hkl

Additional supplementary materials:  crystallographic information; 3D view; checkCIF report

## Figures and Tables

**Table 1:** Hydrogen-bond geometry (Å, °)

*D*—H⋯*A*	*D*—H	H⋯*A*	*D*⋯*A*	*D*—H⋯*A*
O2—H201⋯O1^i^	0.82	1.98	2.793 (4)	169

Symmetry code: (i) 
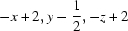
.

**Fig. 1.**
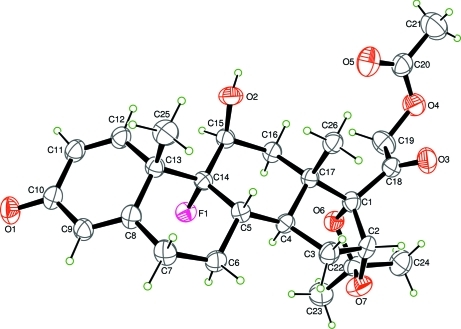
Molecular structure of the title compound (I) showing atom-labelling scheme and displacement ellipsoids at 50% probability level. H atoms are shown as small circles of arbitrary radii.

**Fig. 2.**
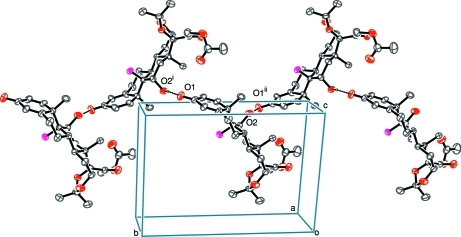
A chain of molecules linked through hydrogen-bonded interactions propagating along b axis. Hydrogen bonds are shown as dashed lines. [Symmetric code: (i)(2 - x,-1/2 + y,2 - z); (ii)(2 - x,1/2 + y,2 - z)]

## supplementary crystallographic information

### Comment

The glucocorticoid triamcinolone acetonide is clinically used for the treatment of suppression of inflammation in chronic inflammatory diseases such as asthma, rheumatiod arthritis, inflammatory bowel disease and autoimmune diseases (Barnes, 1998; Buttgereit, 2000; Uckermann *et al.*, 2005). Triamcinolone acetonide acetate is another derivatives of triamcinolone, which has been used in therapy for several decades. Despite its great importance, no crystal structures of the title compound has been reported. But crystal structure of it's analogue compounds, triamcinolone diacetate chloroform solvate and triamcinole acetonide methanol solvate have been reported (Suitchlmezian *et al.*, 2006 & Jess *et al.*, 2006). In the title compound, the bond lengths and angles are in agreement with those reported for other triamcinolone derivatives (Suitchlmezian *et al.*, 2006; Jess *et al.*, 2006). and are within the expected ranges. The molecular framework consists of five condensed rings, including three six-membered rings and two five-membered rings. Atom O1 is coplanar with cyclohexa-1,4-diene ring (C8—C9—C10—C11—C12—C13), which is planar. Two central six-membered rings (C5—C6—C7—C8—C13—C14) and (C4—C5—C14—C15—C16—C17) are in chair conformation. Two five-membered rings display twisted envelope conformations. The respective r.m.s. deviations for four atoms C1/C2/C3/C17 and C1/C2/O6/O7 are 0.0359 Å, 0.0225Å respectively. The fifth atoms C4, C22 deviate from the above planes by 0.688 (4) Å, 0.458 (4)Å respectively. Hydroxy group and one of the carboxyl O atom are involved in the hydrogen- bonded network. Atom O2 from hydroxy group in the molecule at (*x*,*y*,*z*) act as hydrogen bond donor, to O1 atom of carboxyl group in the molecule (2 - *x*,-1/2 + *y*,2 - *z*). Crystal packing is influenced by this intermolecular hydrogen bond interaction that links the molecules into a chain propagating along *b* axis.

### Experimental

The crude product of the title compound was supplied by Zhejiang Xianju Pharmaceutical Co., LTD. It was recrystallized from methanol solution, giving single crystals suitable for X-ray diffraction.

### Refinement

H atoms were placed in calculated positions with C—H = 0.93–0.98 and O—H = 0.82 Å, and included in the refinement in riding model with *U*_iso_(H) = 1.2*U*_eq_ or 1.5*U*_eq_(carrier atom).

### Crystal data

**Table:**

C_26_H_33_FO_7_	*F*(000) = 508
*M**_r_* = 476.52	*D*_x_ = 1.301 Mg m^−^^3^
Monoclinic, *P*2_1_	Mo *K*α radiation, λ = 0.71073 Å
Hall symbol: P 2yb	Cell parameters from 8589 reflections
*a* = 7.5460 (7) Å	θ = 3.2–27.4°
*b* = 14.8102 (4) Å	µ = 0.10 mm^−^^1^
*c* = 11.5773 (3) Å	*T* = 296 K
β = 109.905 (1)°	Chunk, colorless
*V* = 1216.56 (12) Å^3^	0.38 × 0.33 × 0.26 mm
*Z* = 2	

### Data collection

**Table:**

Rigaku R-AXIS RAPID/ZJUG diffractometer	1866 reflections with *I* > 2σ(*I*)
Radiation source: rolling anode	*R*_int_ = 0.027
graphite	θ_max_ = 25.0°, θ_min_ = 3.2°
Detector resolution: 10.00 pixels mm^-1^	*h* = −8→8
ω scans	*k* = −17→17
9347 measured reflections	*l* = −13→13
2216 independent reflections	

### Refinement

**Table:**

Refinement on *F*^2^	Primary atom site location: structure-invariant direct methods
Least-squares matrix: full	Secondary atom site location: difference Fourier map
*R*[*F*^2^ > 2σ(*F*^2^)] = 0.035	Hydrogen site location: inferred from neighbouring sites
*wR*(*F*^2^) = 0.100	H-atom parameters constrained
*S* = 1.00	*w* = 1/[σ^2^(*F*_o_^2^) + (0.0428*P*)^2^ + 0.6583*P*] where *P* = (*F*_o_^2^ + 2*F*_c_^2^)/3
2216 reflections	(Δ/σ)_max_ < 0.001
308 parameters	Δρ_max_ = 0.18 e Å^−^^3^
1 restraint	Δρ_min_ = −0.21 e Å^−^^3^

### Special details

**Table:**

Geometry. All e.s.d.'s (except the e.s.d. in the dihedral angle between two l.s. planes) are estimated using the full covariance matrix. The cell e.s.d.'s are taken into account individually in the estimation of e.s.d.'s in distances, angles and torsion angles; correlations between e.s.d.'s in cell parameters are only used when they are defined by crystal symmetry. An approximate (isotropic) treatment of cell e.s.d.'s is used for estimating e.s.d.'s involving l.s. planes.
Refinement. Refinement of *F*^2^ against ALL reflections. The weighted *R*-factor *wR* and goodness of fit *S* are based on *F*^2^, conventional *R*-factors *R* are based on *F*, with *F* set to zero for negative *F*^2^. The threshold expression of *F*^2^ > σ(*F*^2^) is used only for calculating *R*-factors(gt) *etc*. and is not relevant to the choice of reflections for refinement. *R*-factors based on *F*^2^ are statistically about twice as large as those based on *F*, and *R*- factors based on ALL data will be even larger.

### Fractional atomic coordinates and isotropic or equivalent isotropic displacement parameters (Å^2^)

**Table:**

	*x*	*y*	*z*	*U*_iso_*/*U*_eq_	
F1	0.6313 (3)	0.54106 (13)	0.76554 (17)	0.0388 (5)	
O6	0.5255 (4)	0.35878 (18)	0.4342 (2)	0.0425 (6)	
O7	0.2113 (4)	0.3480 (2)	0.3882 (2)	0.0580 (8)	
O2	0.9010 (4)	0.3514 (2)	0.9117 (2)	0.0498 (7)	
H201	0.9978	0.3303	0.9056	0.075*	
O1	0.7606 (4)	0.76945 (19)	1.0779 (3)	0.0558 (8)	
O4	0.8680 (4)	0.1362 (2)	0.4470 (3)	0.0638 (9)	
O3	0.5322 (4)	0.1277 (2)	0.4898 (3)	0.0582 (8)	
O5	0.9888 (5)	0.1500 (2)	0.6510 (3)	0.0728 (9)	
C8	0.5342 (5)	0.5590 (3)	0.9699 (3)	0.0407 (9)	
C14	0.6558 (5)	0.4623 (2)	0.8393 (3)	0.0322 (7)	
C16	0.7704 (5)	0.3720 (3)	0.6873 (3)	0.0376 (8)	
H16A	0.7556	0.4198	0.6275	0.045*	
H16B	0.8731	0.3336	0.6848	0.045*	
C5	0.4670 (5)	0.4121 (2)	0.7923 (3)	0.0336 (8)	
H5	0.4738	0.3602	0.8461	0.040*	
C7	0.3424 (5)	0.5160 (3)	0.9231 (4)	0.0480 (10)	
H7A	0.2468	0.5613	0.9171	0.058*	
H7B	0.3334	0.4700	0.9805	0.058*	
C13	0.6966 (5)	0.4988 (2)	0.9729 (3)	0.0361 (8)	
C17	0.5885 (5)	0.3160 (2)	0.6510 (3)	0.0333 (8)	
C9	0.5589 (5)	0.6453 (3)	1.0052 (3)	0.0433 (9)	
H9	0.4539	0.6789	1.0033	0.052*	
C6	0.3070 (5)	0.4733 (3)	0.7971 (4)	0.0425 (9)	
H6A	0.1915	0.4384	0.7748	0.051*	
H6B	0.2891	0.5210	0.7367	0.051*	
C4	0.4318 (4)	0.3771 (3)	0.6626 (3)	0.0339 (8)	
H4	0.4259	0.4300	0.6104	0.041*	
C10	0.7421 (5)	0.6892 (3)	1.0465 (3)	0.0424 (9)	
C11	0.9034 (5)	0.6337 (3)	1.0487 (4)	0.0479 (10)	
H11	1.0234	0.6590	1.0748	0.058*	
C3	0.2545 (5)	0.3219 (3)	0.6011 (4)	0.0450 (9)	
H3A	0.1427	0.3595	0.5788	0.054*	
H3B	0.2417	0.2735	0.6542	0.054*	
C15	0.8226 (5)	0.4144 (3)	0.8163 (3)	0.0368 (8)	
H15	0.9191	0.4603	0.8229	0.044*	
C12	0.8813 (5)	0.5480 (3)	1.0142 (3)	0.0432 (9)	
H12	0.9880	0.5160	1.0156	0.052*	
C26	0.6145 (5)	0.2300 (3)	0.7297 (3)	0.0430 (9)	
H26A	0.7140	0.1940	0.7194	0.065*	
H26B	0.4995	0.1959	0.7042	0.065*	
H26C	0.6464	0.2464	0.8146	0.065*	
C19	0.7634 (7)	0.2173 (3)	0.4445 (4)	0.0599 (11)	
H19A	0.7220	0.2420	0.3620	0.072*	
H19B	0.8453	0.2614	0.4989	0.072*	
C1	0.5043 (6)	0.2880 (3)	0.5130 (3)	0.0386 (8)	
C2	0.2878 (5)	0.2844 (3)	0.4866 (3)	0.0447 (9)	
H2	0.2359	0.2235	0.4651	0.054*	
C22	0.3498 (6)	0.3674 (3)	0.3347 (3)	0.0502 (10)	
C18	0.5929 (6)	0.2019 (3)	0.4831 (3)	0.0438 (9)	
C25	0.7128 (7)	0.4231 (3)	1.0695 (4)	0.0530 (10)	
H25A	0.5968	0.3898	1.0471	0.080*	
H25B	0.7376	0.4499	1.1490	0.080*	
H25C	0.8140	0.3830	1.0720	0.080*	
C24	0.3353 (8)	0.3027 (4)	0.2306 (4)	0.0689 (14)	
H24A	0.4307	0.3168	0.1959	0.103*	
H24B	0.2132	0.3081	0.1685	0.103*	
H24C	0.3527	0.2419	0.2616	0.103*	
C20	0.9797 (6)	0.1098 (3)	0.5594 (4)	0.0568 (11)	
C23	0.3293 (8)	0.4648 (4)	0.2940 (4)	0.0709 (14)	
H23A	0.4223	0.4789	0.2572	0.106*	
H23B	0.3469	0.5032	0.3639	0.106*	
H23C	0.2057	0.4743	0.2352	0.106*	
C21	1.0917 (8)	0.0281 (4)	0.5528 (6)	0.0910 (18)	
H21A	1.0576	0.0087	0.4689	0.137*	
H21B	1.0658	−0.0194	0.6012	0.137*	
H21C	1.2235	0.0425	0.5840	0.137*	

### Atomic displacement parameters (Å^2^)

**Table:**

	*U*^11^	*U*^22^	*U*^33^	*U*^12^	*U*^13^	*U*^23^
F1	0.0395 (11)	0.0373 (12)	0.0392 (11)	−0.0020 (9)	0.0130 (9)	0.0066 (9)
O6	0.0465 (14)	0.0450 (16)	0.0341 (12)	−0.0009 (12)	0.0114 (11)	0.0025 (12)
O7	0.0448 (15)	0.077 (2)	0.0448 (14)	0.0049 (15)	0.0062 (12)	0.0030 (15)
O2	0.0450 (15)	0.0604 (19)	0.0387 (13)	0.0179 (14)	0.0076 (12)	0.0010 (13)
O1	0.0597 (18)	0.0436 (18)	0.0664 (19)	−0.0124 (14)	0.0245 (15)	−0.0184 (15)
O4	0.0615 (18)	0.068 (2)	0.0601 (18)	0.0153 (16)	0.0186 (15)	−0.0178 (16)
O3	0.069 (2)	0.0429 (18)	0.0593 (17)	−0.0048 (15)	0.0181 (15)	−0.0110 (14)
O5	0.082 (2)	0.075 (2)	0.0631 (19)	−0.0134 (19)	0.0274 (17)	−0.0146 (19)
C8	0.039 (2)	0.046 (2)	0.043 (2)	−0.0073 (17)	0.0224 (16)	−0.0088 (18)
C14	0.0344 (18)	0.0307 (18)	0.0322 (16)	0.0007 (15)	0.0123 (14)	0.0025 (15)
C16	0.0355 (18)	0.045 (2)	0.0375 (18)	−0.0023 (17)	0.0195 (15)	−0.0066 (17)
C5	0.0340 (17)	0.0343 (19)	0.0372 (17)	−0.0021 (15)	0.0183 (15)	−0.0010 (15)
C7	0.039 (2)	0.054 (3)	0.062 (2)	−0.0047 (19)	0.032 (2)	−0.013 (2)
C13	0.0371 (19)	0.038 (2)	0.0347 (18)	−0.0003 (16)	0.0142 (16)	−0.0025 (15)
C17	0.0349 (18)	0.0345 (19)	0.0324 (16)	−0.0015 (15)	0.0140 (15)	−0.0026 (15)
C9	0.0369 (19)	0.047 (2)	0.048 (2)	−0.0009 (17)	0.0170 (17)	−0.0122 (18)
C6	0.0271 (17)	0.050 (2)	0.053 (2)	−0.0051 (16)	0.0169 (16)	−0.0100 (19)
C4	0.0316 (17)	0.0364 (19)	0.0345 (17)	−0.0037 (15)	0.0122 (15)	−0.0007 (15)
C10	0.042 (2)	0.048 (3)	0.0382 (19)	−0.0085 (18)	0.0159 (17)	−0.0087 (18)
C11	0.0365 (19)	0.057 (3)	0.049 (2)	−0.0062 (19)	0.0123 (17)	−0.013 (2)
C3	0.037 (2)	0.048 (2)	0.052 (2)	−0.0076 (17)	0.0171 (17)	−0.0077 (18)
C15	0.0343 (18)	0.038 (2)	0.0406 (19)	0.0000 (16)	0.0163 (15)	−0.0046 (16)
C12	0.0379 (19)	0.053 (3)	0.0385 (19)	−0.0013 (18)	0.0130 (16)	−0.0100 (19)
C26	0.046 (2)	0.042 (2)	0.0373 (19)	−0.0022 (18)	0.0098 (17)	−0.0018 (18)
C19	0.068 (3)	0.057 (3)	0.064 (3)	0.011 (2)	0.035 (2)	−0.004 (2)
C1	0.0438 (19)	0.038 (2)	0.0345 (18)	−0.0009 (17)	0.0139 (16)	−0.0025 (16)
C2	0.044 (2)	0.045 (2)	0.041 (2)	−0.0104 (18)	0.0103 (17)	−0.0103 (17)
C22	0.048 (2)	0.062 (3)	0.0328 (18)	0.006 (2)	0.0041 (17)	0.0013 (19)
C18	0.047 (2)	0.041 (2)	0.0375 (19)	0.0025 (18)	0.0079 (17)	−0.0035 (17)
C25	0.066 (3)	0.058 (3)	0.038 (2)	0.006 (2)	0.0218 (19)	0.0061 (19)
C24	0.079 (3)	0.075 (3)	0.045 (2)	−0.001 (3)	0.012 (2)	−0.011 (2)
C20	0.047 (2)	0.054 (3)	0.069 (3)	−0.008 (2)	0.018 (2)	−0.009 (2)
C23	0.084 (4)	0.060 (3)	0.055 (3)	0.011 (3)	0.004 (3)	0.009 (2)
C21	0.080 (4)	0.069 (4)	0.123 (5)	0.012 (3)	0.033 (4)	−0.008 (4)

### Geometric parameters (Å, °)

**Table:**

F1—C14	1.420 (4)	C6—H6B	0.9700
O6—C1	1.434 (4)	C4—C3	1.523 (5)
O6—C22	1.436 (4)	C4—H4	0.9800
O7—C22	1.414 (5)	C10—C11	1.462 (6)
O7—C2	1.439 (5)	C11—C12	1.325 (6)
O2—C15	1.412 (5)	C11—H11	0.9300
O2—H201	0.8200	C3—C2	1.535 (5)
O1—C10	1.237 (5)	C3—H3A	0.9700
O4—C20	1.346 (5)	C3—H3B	0.9700
O4—C19	1.431 (5)	C15—H15	0.9800
O3—C18	1.204 (5)	C12—H12	0.9300
O5—C20	1.197 (5)	C26—H26A	0.9600
C8—C9	1.335 (5)	C26—H26B	0.9600
C8—C7	1.503 (5)	C26—H26C	0.9600
C8—C13	1.506 (5)	C19—C18	1.516 (6)
C14—C5	1.533 (5)	C19—H19A	0.9700
C14—C15	1.545 (5)	C19—H19B	0.9700
C14—C13	1.567 (5)	C1—C18	1.533 (5)
C16—C17	1.535 (5)	C1—C2	1.557 (6)
C16—C15	1.542 (5)	C2—H2	0.9800
C16—H16A	0.9700	C22—C23	1.509 (7)
C16—H16B	0.9700	C22—C24	1.514 (6)
C5—C4	1.523 (5)	C25—H25A	0.9600
C5—C6	1.526 (5)	C25—H25B	0.9600
C5—H5	0.9800	C25—H25C	0.9600
C7—C6	1.528 (5)	C24—H24A	0.9600
C7—H7A	0.9700	C24—H24B	0.9600
C7—H7B	0.9700	C24—H24C	0.9600
C13—C12	1.499 (5)	C20—C21	1.493 (7)
C13—C25	1.559 (5)	C23—H23A	0.9600
C17—C4	1.531 (5)	C23—H23B	0.9600
C17—C26	1.539 (5)	C23—H23C	0.9600
C17—C1	1.561 (5)	C21—H21A	0.9600
C9—C10	1.453 (5)	C21—H21B	0.9600
C9—H9	0.9300	C21—H21C	0.9600
C6—H6A	0.9700		
			
C1—O6—C22	107.6 (3)	O2—C15—C16	112.9 (3)
C22—O7—C2	108.9 (3)	O2—C15—C14	108.5 (3)
C15—O2—H201	109.5	C16—C15—C14	113.5 (3)
C20—O4—C19	115.0 (3)	O2—C15—H15	107.2
C9—C8—C7	122.2 (4)	C16—C15—H15	107.2
C9—C8—C13	122.1 (3)	C14—C15—H15	107.2
C7—C8—C13	115.7 (3)	C11—C12—C13	124.7 (4)
F1—C14—C5	105.6 (3)	C11—C12—H12	117.6
F1—C14—C15	102.7 (2)	C13—C12—H12	117.6
C5—C14—C15	115.4 (3)	C17—C26—H26A	109.5
F1—C14—C13	104.6 (3)	C17—C26—H26B	109.5
C5—C14—C13	111.4 (3)	H26A—C26—H26B	109.5
C15—C14—C13	115.5 (3)	C17—C26—H26C	109.5
C17—C16—C15	113.3 (3)	H26A—C26—H26C	109.5
C17—C16—H16A	108.9	H26B—C26—H26C	109.5
C15—C16—H16A	108.9	O4—C19—C18	112.7 (4)
C17—C16—H16B	108.9	O4—C19—H19A	109.1
C15—C16—H16B	108.9	C18—C19—H19A	109.1
H16A—C16—H16B	107.7	O4—C19—H19B	109.1
C4—C5—C6	111.3 (3)	C18—C19—H19B	109.1
C4—C5—C14	110.1 (2)	H19A—C19—H19B	107.8
C6—C5—C14	110.5 (3)	O6—C1—C18	108.2 (3)
C4—C5—H5	108.3	O6—C1—C2	103.8 (3)
C6—C5—H5	108.3	C18—C1—C2	115.9 (3)
C14—C5—H5	108.3	O6—C1—C17	111.3 (3)
C8—C7—C6	110.7 (3)	C18—C1—C17	113.3 (3)
C8—C7—H7A	109.5	C2—C1—C17	104.1 (3)
C6—C7—H7A	109.5	O7—C2—C3	107.9 (3)
C8—C7—H7B	109.5	O7—C2—C1	104.1 (3)
C6—C7—H7B	109.5	C3—C2—C1	106.7 (3)
H7A—C7—H7B	108.1	O7—C2—H2	112.5
C12—C13—C8	112.4 (3)	C3—C2—H2	112.5
C12—C13—C25	106.5 (3)	C1—C2—H2	112.5
C8—C13—C25	107.9 (3)	O7—C22—O6	104.4 (3)
C12—C13—C14	109.3 (3)	O7—C22—C23	108.6 (4)
C8—C13—C14	107.2 (3)	O6—C22—C23	107.7 (4)
C25—C13—C14	113.7 (3)	O7—C22—C24	110.8 (4)
C4—C17—C16	107.5 (3)	O6—C22—C24	112.2 (4)
C4—C17—C26	112.6 (3)	C23—C22—C24	112.7 (4)
C16—C17—C26	111.3 (3)	O3—C18—C19	122.4 (4)
C4—C17—C1	100.9 (3)	O3—C18—C1	122.7 (4)
C16—C17—C1	116.0 (3)	C19—C18—C1	114.9 (4)
C26—C17—C1	108.2 (3)	C13—C25—H25A	109.5
C8—C9—C10	123.2 (4)	C13—C25—H25B	109.5
C8—C9—H9	118.4	H25A—C25—H25B	109.5
C10—C9—H9	118.4	C13—C25—H25C	109.5
C5—C6—C7	113.4 (3)	H25A—C25—H25C	109.5
C5—C6—H6A	108.9	H25B—C25—H25C	109.5
C7—C6—H6A	108.9	C22—C24—H24A	109.5
C5—C6—H6B	108.9	C22—C24—H24B	109.5
C7—C6—H6B	108.9	H24A—C24—H24B	109.5
H6A—C6—H6B	107.7	C22—C24—H24C	109.5
C3—C4—C5	118.3 (3)	H24A—C24—H24C	109.5
C3—C4—C17	102.9 (3)	H24B—C24—H24C	109.5
C5—C4—C17	114.0 (3)	O5—C20—O4	122.7 (4)
C3—C4—H4	107.0	O5—C20—C21	125.8 (5)
C5—C4—H4	107.0	O4—C20—C21	111.4 (4)
C17—C4—H4	107.0	C22—C23—H23A	109.5
O1—C10—C9	121.8 (4)	C22—C23—H23B	109.5
O1—C10—C11	121.7 (4)	H23A—C23—H23B	109.5
C9—C10—C11	116.6 (3)	C22—C23—H23C	109.5
C12—C11—C10	121.0 (4)	H23A—C23—H23C	109.5
C12—C11—H11	119.5	H23B—C23—H23C	109.5
C10—C11—H11	119.5	C20—C21—H21A	109.5
C4—C3—C2	102.9 (3)	C20—C21—H21B	109.5
C4—C3—H3A	111.2	H21A—C21—H21B	109.5
C2—C3—H3A	111.2	C20—C21—H21C	109.5
C4—C3—H3B	111.2	H21A—C21—H21C	109.5
C2—C3—H3B	111.2	H21B—C21—H21C	109.5
H3A—C3—H3B	109.1		
			
F1—C14—C5—C4	67.2 (3)	F1—C14—C15—O2	161.5 (3)
C15—C14—C5—C4	−45.5 (4)	C5—C14—C15—O2	−84.1 (3)
C13—C14—C5—C4	−179.8 (3)	C13—C14—C15—O2	48.3 (4)
F1—C14—C5—C6	−56.1 (3)	F1—C14—C15—C16	−72.2 (4)
C15—C14—C5—C6	−168.9 (3)	C5—C14—C15—C16	42.2 (4)
C13—C14—C5—C6	56.9 (4)	C13—C14—C15—C16	174.6 (3)
C9—C8—C7—C6	125.6 (4)	C10—C11—C12—C13	−1.3 (6)
C13—C8—C7—C6	−53.7 (5)	C8—C13—C12—C11	2.3 (5)
C9—C8—C13—C12	−2.4 (5)	C25—C13—C12—C11	−115.6 (4)
C7—C8—C13—C12	176.9 (3)	C14—C13—C12—C11	121.2 (4)
C9—C8—C13—C25	114.7 (4)	C20—O4—C19—C18	−77.7 (5)
C7—C8—C13—C25	−66.0 (4)	C22—O6—C1—C18	99.8 (3)
C9—C8—C13—C14	−122.5 (4)	C22—O6—C1—C2	−23.8 (4)
C7—C8—C13—C14	56.8 (4)	C22—O6—C1—C17	−135.2 (3)
F1—C14—C13—C12	−65.8 (3)	C4—C17—C1—O6	78.1 (3)
C5—C14—C13—C12	−179.5 (3)	C16—C17—C1—O6	−37.7 (4)
C15—C14—C13—C12	46.3 (4)	C26—C17—C1—O6	−163.5 (3)
F1—C14—C13—C8	56.3 (3)	C4—C17—C1—C18	−159.7 (3)
C5—C14—C13—C8	−57.4 (4)	C16—C17—C1—C18	84.5 (4)
C15—C14—C13—C8	168.4 (3)	C26—C17—C1—C18	−41.3 (4)
F1—C14—C13—C25	175.4 (3)	C4—C17—C1—C2	−33.0 (4)
C5—C14—C13—C25	61.8 (4)	C16—C17—C1—C2	−148.8 (3)
C15—C14—C13—C25	−72.5 (4)	C26—C17—C1—C2	85.4 (3)
C15—C16—C17—C4	55.6 (4)	C22—O7—C2—C3	129.1 (3)
C15—C16—C17—C26	−68.1 (4)	C22—O7—C2—C1	16.0 (4)
C15—C16—C17—C1	167.7 (3)	C4—C3—C2—O7	−90.1 (3)
C7—C8—C9—C10	−177.7 (4)	C4—C3—C2—C1	21.3 (4)
C13—C8—C9—C10	1.6 (6)	O6—C1—C2—O7	4.9 (4)
C4—C5—C6—C7	−175.6 (3)	C18—C1—C2—O7	−113.5 (3)
C14—C5—C6—C7	−52.9 (4)	C17—C1—C2—O7	121.5 (3)
C8—C7—C6—C5	50.0 (5)	O6—C1—C2—C3	−109.0 (3)
C6—C5—C4—C3	−59.5 (4)	C18—C1—C2—C3	132.6 (3)
C14—C5—C4—C3	177.6 (3)	C17—C1—C2—C3	7.5 (4)
C6—C5—C4—C17	179.4 (3)	C2—O7—C22—O6	−31.0 (4)
C14—C5—C4—C17	56.5 (4)	C2—O7—C22—C23	−145.7 (3)
C16—C17—C4—C3	169.2 (3)	C2—O7—C22—C24	90.0 (4)
C26—C17—C4—C3	−67.9 (4)	C1—O6—C22—O7	34.3 (4)
C1—C17—C4—C3	47.2 (3)	C1—O6—C22—C23	149.6 (3)
C16—C17—C4—C5	−61.5 (4)	C1—O6—C22—C24	−85.8 (4)
C26—C17—C4—C5	61.5 (4)	O4—C19—C18—O3	−14.4 (6)
C1—C17—C4—C5	176.6 (3)	O4—C19—C18—C1	165.5 (3)
C8—C9—C10—O1	179.5 (4)	O6—C1—C18—O3	−146.6 (4)
C8—C9—C10—C11	−0.4 (6)	C2—C1—C18—O3	−30.6 (5)
O1—C10—C11—C12	−179.7 (4)	C17—C1—C18—O3	89.6 (5)
C9—C10—C11—C12	0.2 (6)	O6—C1—C18—C19	33.5 (4)
C5—C4—C3—C2	−169.4 (3)	C2—C1—C18—C19	149.4 (3)
C17—C4—C3—C2	−42.8 (4)	C17—C1—C18—C19	−90.4 (4)
C17—C16—C15—O2	76.4 (4)	C19—O4—C20—O5	1.2 (6)
C17—C16—C15—C14	−47.6 (4)	C19—O4—C20—C21	−176.7 (4)

### Hydrogen-bond geometry (Å, °)

**Table:**

*D*—H···*A*	*D*—H	H···*A*	*D*···*A*	*D*—H···*A*
O2—H201···O1^i^	0.82	1.98	2.793 (4)	169

Symmetry codes: (i) −*x*+2, *y*−1/2, −*z*+2.
